# Intermittent fasting and continuous energy restriction result in similar changes in body composition and muscle strength when combined with a 12 week resistance training program

**DOI:** 10.1007/s00394-022-02804-3

**Published:** 2022-01-27

**Authors:** Stephen J. Keenan, Matthew B. Cooke, Ebrahim Bani Hassan, Won Sun Chen, Josef Sullivan, Sam X. Wu, Doa El-Ansary, Mahdi Imani, Regina Belski

**Affiliations:** 1 Faculty of Health, Arts and Design, School of Health Sciences, Swinburne University of Technology, John Street, Hawthorn, Melbourne, VIC 3122 Australia; 2 Australian Institute for Musculoskeletal Science (AIMSS), The University of Melbourne, Melbourne, VIC Australia; 3 Department of Medicine, Western Health, Melbourne Medical School, The University of Melbourne, Melbourne, VIC Australia; 4 Department of Surgery, Melbourne Medical School, The University of Melbourne, Melbourne, VIC Australia

**Keywords:** Intermittent fasting, Continuous energy restriction, Resistance training, Body composition, Lean body mass, Weight loss

## Abstract

**Purpose:**

The objective of this study was to compare the effects of 12 weeks of resistance training combined with either 5:2 intermittent fasting or continuous energy restriction on body composition, muscle size and quality, and upper and lower body strength.

**Methods:**

Untrained individuals undertook 12 weeks of resistance training plus either continuous energy restriction [20% daily energy restriction (CERT)] or 5:2 intermittent fasting [~ 70% energy restriction 2 days/week, euenergetic consumption 5 days/week (IFT)], with both groups prescribed a mean of ≥ 1.4 g of protein per kilogram of body weight per day. Participants completed 2 supervised resistance and 1 unsupervised aerobic/resistance training combination session per week. Changes in lean body mass (LBM), thigh muscle size and quality, strength and dietary intake were assessed.

**Results:**

Thirty-four participants completed the study (CERT = 17, IFT = 17). LBM was significantly increased (+ 3.7%, *p* < 0.001) and body weight (− 4.6%, *p* < 0.001) and fat (− 24.1%, *p* < 0.001) were significantly reduced with no significant difference between groups, though results differed by sex. Both groups showed improvements in thigh muscle size and quality, and reduced intramuscular and subcutaneous fat assessed by ultrasonography and peripheral quantitative computed tomography (pQCT), respectively. The CERT group demonstrated a significant increase in muscle surface area assessed by pQCT compared to the IFT group. Similar gains in upper and lower body strength and muscular endurance were observed between groups.

**Conclusion:**

When combined with resistance training and moderate protein intake, continuous energy restriction and 5:2 intermittent fasting resulted in similar improvements in body composition, muscle quality, and strength. ACTRN: ACTRN12620000920998, September 2020, retrospectively registered.

**Supplementary Information:**

The online version contains supplementary material available at 10.1007/s00394-022-02804-3.

## Introduction

Energy-restricted diets are becoming increasingly popular amongst individuals for a variety of reasons, including improving body composition and general health and wellbeing. Regardless of the reason, these diets commonly lead to weight loss in the form of both fat and lean body mass (LBM) [1]. While fat loss is usually desirable, reductions in skeletal muscle mass (a major component of LBM) may lead to a number of deleterious short- and long-term consequences, such as hyperphagia and reduced basal metabolic rate, which may compromise long term weight loss success [2]; increased risk of strength loss and disability, especially in older adults [3, 4]; and potential metabolic issues incumbent with low muscle mass [5].

The mechanisms behind weight-loss-induced reductions in LBM are not fully understood, however; the impact of energy restriction on protein turnover and net muscle protein balance may be a contributing factor [6]. Skeletal muscle mass is determined by a balance between muscle protein synthesis (MPS) and muscle protein breakdown (MPB), which remains equal during energy balance [7]. Conversely, during short-term, continuous energy restriction, both post-prandial and post-absorptive MPS are reduced [8], which may lead to an overall negative protein balance, higher protein catabolism to supply amino acids and reductions in muscle mass [6]. Whether this also occurs over extended periods of energy restriction is unclear [9]. Notwithstanding, higher protein intakes, and/or performing resistance training have been shown to partially or completely attenuate these reductions in MPS [6, 10]. Moreover, these strategies, as well as others including slower rates of weight loss, have been utilised to successfully mitigate LBM loss during longer periods of energy restriction [1, 11].

Recently, interest in how the pattern of energy restriction affects changes in LBM during weight loss has increased. Compared to traditional energy-restricted diets that are characterised by moderate daily energy restriction (i.e. continuous energy restriction), alternative patterns such as intermittent fasting where periods of severe energy restriction are interspersed with regular dietary or ad libitum consumption might provide greater protection against LBM loss. Although evidence to support this is currently lacking [12], it seems plausible that short-term periods of energy balance or surplus, especially in close proximity to resistance training, may promote greater maintenance and accrual of LBM than continuous periods of energy restriction/deficit. This could be via greater output during training due to increased energy availability, or differences in acute changes in MPS and anabolic hormonal responses during periods of energy balance/surplus compared to energy deficit [8, 13].

A popular variation of intermittent fasting is the 5:2 fasting diet, which generally involves 2 days per week of severe (consumption of ~ 1600–3000 kJ/day) or complete energy restriction, paired with 5 days of ad libitum or euenergetic consumption [14, 15]. Despite widespread public popularity of the 5:2 intermittent fasting model, little research has investigated its effects on LBM compared to a continuous energy restriction diet, especially when combined with a resistance training program and higher protein intake [16]. Moreover, only a handful of intermittent fasting studies have utilised more sensitive assessments of muscle hypertrophy (e.g. ultrasonography) to simultaneously assess changes in muscle growth [17–20]. Thus, the purpose of this study was to investigate and compare the effects of 12 weeks of resistance training combined with either a 5:2 intermittent fasting or continuous energy restriction style diet matched for energy and protein intake on body composition (especially LBM), indicators of muscle hypertrophy and quality, and upper and lower body strength. The primary outcome of this study was a change in LBM. It was hypothesised that 5:2 intermittent fasting would result in maintenance or greater accrual of LBM compared with continuous energy restriction.

## Methods

### Participants

Participants were recruited through advertising on social media channels targeting current and past university students in Victoria, Australia. A total of 194 individuals responded to the advertisement and underwent initial screening. Only 44 were deemed eligible and were recruited for the study. Participants were eligible for inclusion if they: (i) were aged between 18 and 45 years; (ii) had a body mass index (BMI) of 22.0–35.0 kg/m^2^; (iii) had a body fat percentage > 18% for males or > 25% for females as measured via dual x-ray absorptiometry (DXA); (iv) had not followed a structured resistance training program in the previous 6 months and; (v) had been weight stable for 3 months prior to the study (< 5% weight loss or weight gain). Participants were excluded if they: (i) were smokers; (ii) had diabetes; (iii) had a history of cardiovascular disease; (iv) were taking dietary supplements and were unwilling to cease these for the duration of the study; (v) were taking glucose or lipid lowering, or weight loss medication; (vi) had a current physical condition that may have been exacerbated by resistance training as determined by their general practitioner, (vii) were pregnant or intended to become pregnant in the following 3–4 months; (viii) were menopausal or post-menopausal; (ix) had a history of disordered eating; (x) had a current or previous respiratory condition likely to be exacerbated by the intervention; (xi) had a current or previous gastrointestinal disorder likely to be exacerbated by the intervention; (xii) had any allergy to any components of the supplement product to be supplied; (xiii) were unable to commit to fasting on assigned days if randomised to the intermittent fasting group; (xiv) did not speak English at a level at which they were able to understand and complete the requirements of the study or; (xv) disclosed any other chronic disease or condition, or were taking any other medication that investigators deemed would contraindicate the study intervention.

### Randomisation and study overview

**Fig. 1:**
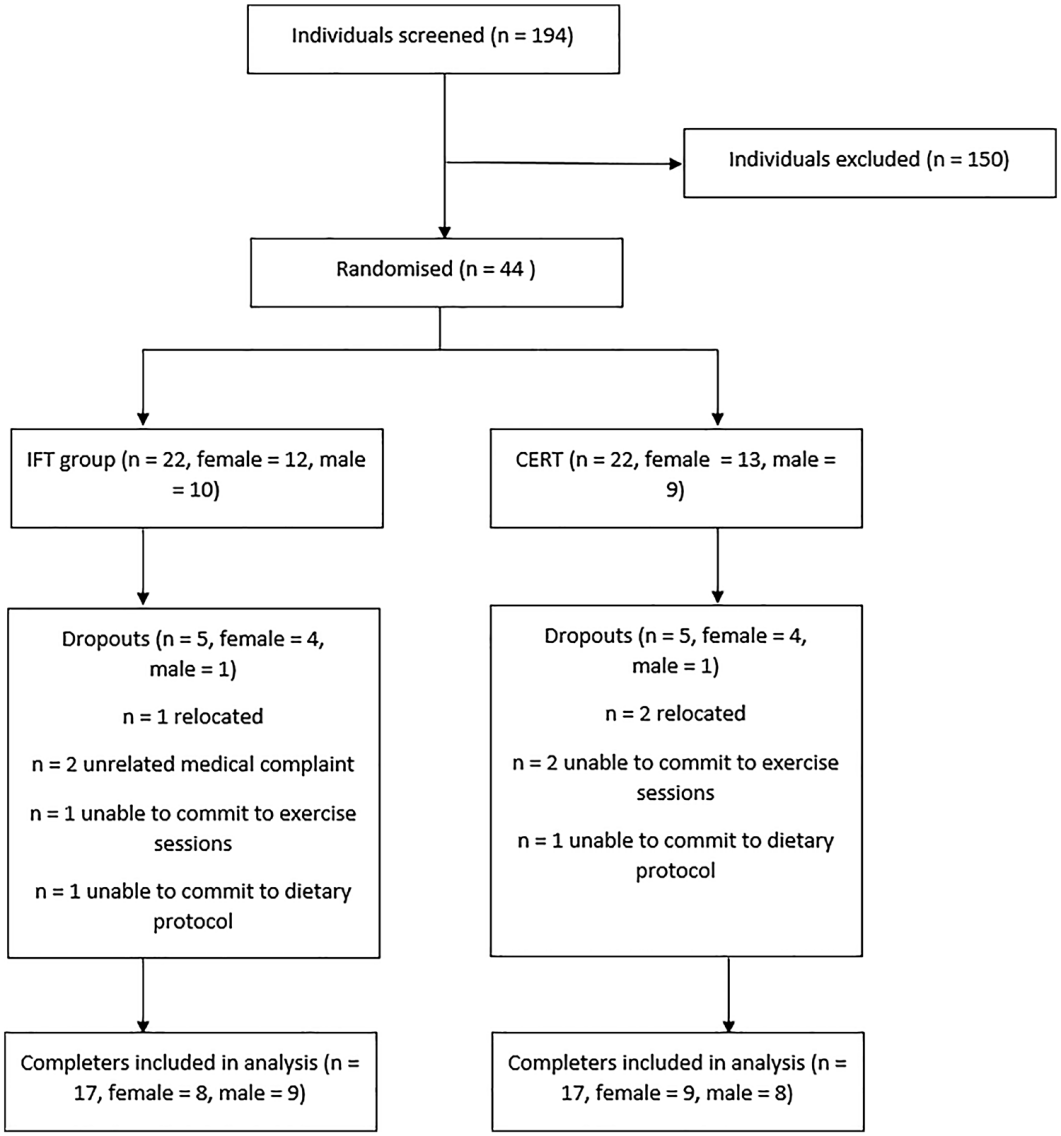
Participant flow diagram

Participants who were eligible for the study were stratified by age, sex and BMI before being randomised by coin toss into either the intermittent fasting plus training (IFT) or continuous energy restriction plus training (CERT) groups for 12 weeks. The random allocation sequence was not explicitly concealed. Participants in the IFT group undertook a 5:2 style-fasting protocol, while those in the CERT group undertook a continuous energy restriction style feeding pattern. Both groups completed supervised resistance training twice per week, and resistance/aerobic combination training once per week. The intervention took place from February to November 2019, spread across 6 groups, starting 2–4 weeks apart. A flow chart showing participant movement through the study can be seen in Fig. 1. This study was approved by the Swinburne University of Technology Human Research Ethics Committee (project #2018/322).

### Diet protocol

Basal energy requirements for all participants were calculated using the Mifflin St. Jeor equation [21], with total energy requirements calculated by applying an activity factor of 1.4 representing a recreational level of activity (based on prescribed exercise). At the beginning of the intervention period, all participants were provided with example meal plans that would result in mean consumption of approximately 80% of estimated energy requirements and 1.4 g of protein per kilogram of body weight per day (g/kg/day) over the 12 week intervention. Meal plans were customised based on food preferences for each individual and provided by a dietitian along with brief education on the Australian healthy eating guidelines [22]. As participants in the IFT group had limited energy available on fasting days to reach recommended protein intakes, they were provided with protein shakes and high protein soups to get as close to daily protein intake recommendations as possible. Participants were also instructed on how to use the Easy Diet Diary (Xyris Software, Australia, 2019) smartphone application to record their food, and substitute other foods of their choosing into the meal plans while maintaining the same energy and protein intake.

### Intermittent fasting diet protocol

All participants in the IFT group were instructed to consume 100% of their energy requirements for 5 days per week (non-fasting days). On two non-consecutive and non-training days, participants consumed approximately 30% of their estimated energy requirements (~ 2100 kJ for females and ~ 2500 kJ for males) (fasting days), consistent with previous research [23, 24]. On fasting days, participants were prescribed a diet consisting of whey-based protein shakes (Formulite), high-protein soups and steamed/raw vegetables. The macronutrient composition of these supplements and the recommended intake on fasting days can be seen in Table 1. On fasting days, participants were asked to consume all energy during a 6 h window between 12.00 pm and 6.00 pm, to ensure an extended fasting period, but also allow flexibility of consumption to promote compliance. Further, they were allowed ad libitum consumption of non-energy providing beverages on fasting days. To match protein intakes across dietary groups as closely as possible, those in the IFT group were instructed to consume approximately 1.5 g/kg/day of protein on non-fasting days, as their fasting day diets only provided ~ 1.1–1.2 g/kg/day. This design resulted in the prescription of a 20% energy deficit and a mean intake of protein of 1.4 g/kg/day, designed to be isoenergetic and isonitrogenous with the CERT group to explore the differences between patterns of dietary intake while controlling for overall intake as much as possible.

**Table 1:** Composition of fasting day meals consumed by IFT group and overall intake on fasting days

	Male	Female
Foods	2 × meal replacement shakes	1.5 × meal replacement shakes
	1 × high protein soup	1 × high protein soup
	150 g raw/steamed vegetables	150 g raw/steamed vegetables
Nutrients		
Energy (kJ/kcal)	2511/597	2080/495
Protein (g)	93.6	76.7
Carbohydrates (g)	30.4	26.3
Fat (g)	10	8

### Continuous energy restriction

Those randomised to the CERT group were instructed to consume ~ 80% of their total energy requirements daily for the duration of the 12 week intervention. This group represented a best practice ‘control’ group, as moderate energy restriction (15–30%) is most commonly recommended for weight loss [25, 26]. Furthermore, participants were also prescribed consumption of 1.4 g/kg/day of protein. Participants in this group also received customised meal plans and the same education as those in the IFT group.

### Exercise protocol

All participants were required to undertake 3 training sessions each week: 2 resistance training sessions and 1 bodyweight aerobic/resistance training combination session. Training frequency of 3 times per week was seen as practical for the untrained participants, allowing adequate time for recovery, but also the stimulus for muscle growth. The two resistance training sessions were conducted at Swinburne University’s Hawthorn campus, and were supervised by an accredited strength and conditioning coach (who was also the study dietitian). The 2 supervised sessions consisted of variations of the following exercises: push-ups, squats, rows, lunges, bicep curls and dips (Supplemental Fig. 1). The exercises utilised were chosen due to their lack of technical difficulty given the untrained population being researched. Participants completed these exercises in a superset style workout, aiming to complete 12–15 repetitions of each exercise. Once participants were able to complete 3 sets of 15 repetitions in any individual exercise, the weight was increased or exercise variation made more difficult, adhering to the principles of progressive overload. The one bodyweight aerobic/resistance training combination session per week was completed by participants at home using bodyweight exercises consisting of planks, mountain climbers, crunches, burpees, lying side toe-touches and hip bridges. These exercises were also completed using a superset format with 2 min breaks, however, were timed instead of counting repetitions. When participants reached their time goal with good form (self-assessed), they were instructed to increase this by 5 s. All exercise sessions were conducted on non-fasting days in the IFT group to promote maximal energy availability and avoid any detrimental effects of fasting on exercise performance.

## Assessments

### Bodyweight and body composition analysis

Weekly weight measurements were taken in light clothing using bioelectrical impedance scales [Multifrequency segmental body composition analyser; MC-780 Tanita Corporation (Tokyo, Japan)]. Lean body mass, fat mass and body fat percentage were assessed utilising dual x-ray absorptiometry [DXA; Hologic Horizon (Bedford MA)] at baseline and after the intervention period, as previously detailed [27]. The DXA was calibrated for bone mineral density, muscle and fat masses on the morning of each assessment in accordance with manufacturer guidelines using spine and whole-body phantoms, respectively. Short and long-term coefficient of variation for these measurements were well within the acceptable level set by the manufacturer (< 5%).

### Ultrasound

Muscle thickness, cross-sectional area (CSA) and muscle quality [echogenicity (EI)], were measured using ultrasound (SonoSite M-Turbo, SonoSite Australasia Pty Ltd, New South Wales, Australia) with a linear array transducer (5–2 MHz) for the rectus femoris (RF) and vastus intermedius (VI) of the non-dominant leg at baseline and post intervention (Supplemental Fig. 2). All images were acquired and analysed by the same technician. A fidelity check of the image acquisition and analysis was conducted by a health professional with over 10 years of experience in ultrasound imaging and analysis. Varying depths were required to obtain full visualisation of the RF in some instances, however, the gain was kept consistent across measurements. Measurements were acquired with participants in a supine position, with their knee in passive extension. Ultrasound gel was applied to the transducer, which was placed perpendicular to the long axis of the anterior thigh, at a distance of two-thirds from the anterior, superior iliac spine to the superior patellar border, consistent with previous studies [28]. Muscle thickness and CSA were measured in real-time with the on-board functions of the M-turbo, utilising the straight line and tracing functions, respectively. Images were also saved onto the on-board hard drive before being transferred onto a personal computer for EI analysis using ImageJ software (NIH, Bethesda, MD) [29]. EI for RF and VI were measured utilising a standard square of 100 × 100 pixels, or where the predefined square did not fit within the cross section of the muscle, the largest square that fit within the anatomic boundaries of the muscle was utilised, a method which has shown good inter-observer reliability regardless of the level of expertise [30].

### Peripheral quantitative computed tomography (pQCT)

pQCT was utilised to measure the surface area of muscle, intramuscular fat and subcutaneous fat at baseline and post intervention (Supplemental Fig. 3). A single 2.5 mm transverse pQCT; (Stratec XCT3000, Stratec Medizintechnik GmbH, Pforzheim, Germany) scan with a voxel size of 0.4 mm was obtained at mid-thigh region of the non-dominant leg. The mid-thigh was defined as midway between the tip of the greater trochanter and medial edge of the tibial plateau, located by deep palpation. The images were exported and further analyzed by Slice-O-Matic^™^ (Tomovision, Montreal, CA) to determine the muscle, intramuscular fat and subcutaneous fat volumes as previously described [31–33]. After visual checks; where due to beam hardening artefacts the tissue was not segmented (“tagged”) optimally, the assignment of individual voxels or small voxel islands were changed into the correct tissue manually, at the discretion of the operator. The pQCT was calibrated using the manufacturer’s phantom daily, with a coefficient of variation of 0.2%, as described previously [34].

All imaging and image analyses were carried out by a single experienced image analysis specialist (EB) or under his direct supervision.

### Strength testing

Strength testing was undertaken prior to the diet intervention and at the end of week 12. A 3 repetition maximum (3RM) test and strength endurance test were performed for both bench press and leg press. While these exercises were not included within the resistance training program, these were chosen as a standardised method of strength testing using similar movements to those included. After a brief 5 min warm up, participants were instructed on correct lifting and breathing techniques before practicing these using submaximal loads for 10–15 repetitions. Weight was gradually added and repetitions reduced to serve as a functional warm up. Participants then completed a set of 3 repetitions at a self-selected weight close to their perceived capacity, followed by a 3 min rest, with weight being continually added to each subsequent attempt. 3RM was recorded as the last successful attempt before form breakdown, or failure to complete the lift without assistance. The 3RM of each participant was determined within 5 attempts. After the 3RM test, participants were allowed a 5 min rest before undergoing a strength endurance test. These were tested in order such that 3RM bench press was followed by the bench press endurance test, and 3RM leg press was followed by the leg press endurance test. Participants were required to complete as many repetitions as possible of each exercise utilising 70% of their estimated 1 repetition maximum (1RM), calculated from the attained 3RM utilising the Brzycki formula [35] {weight lifted/[1.0278 – (0.0278 × repetitions performed)]}. Failure was determined as the first repetition where the participant required assistance. Repetitions where form was considered inadequate were not counted; however, participants were not stopped from completing subsequent repetitions if this occurred. Volume was calculated as the number of repetitions completed at 70% of 1RM multiplied by the weight lifted.

### Dietary intake

Participants were required to keep a 3 day food diary at baseline and in week 1, 6 and 12 using the Easy Diet Diary (Xyris Software, Australia, 2019) phone application. This software has been shown to produce results similar to other, more time-intensive methods of dietary data collection such as 24 h recalls [36]. Participants recorded all food and drink intake on non-consecutive days that included 2 weekdays and 1 weekend day. Food records were kept on non-fasting days for those in the IFT group. On fasting days, participants were asked to note down any extra food or drink consumed, and whether they had consumed their recommended supplements. Intake on these days was estimated from these records.

### Statistical analysis

Changes in LBM were considered the primary outcome of this study. Sample size calculations were conducted using GPower version 3.1 [37] based on previous research showing a 2.4 kg loss of LBM in response to similar study methodology to that of the current study in a comparable population [38]. This was contrasted with research into intermittent fasting plus resistance or endurance training that has shown a minimal effect on LBM, albeit with smaller reductions in weight [16, 23]. It was calculated that using an α error probability of 0.05 and power of 80%, 22 participants would be required to detect a 2.4 kg difference in LBM between groups. Assuming a 33% attrition rate, total estimated sample size requirements were 29 participants. Results are presented as mean (± SD). Normality was assessed using the Shapiro–Wilk test and visual inspection of Q–Q plots. Assumptions of normality were violated for intramuscular fat only. Intramuscular fat was log10 transformed, resulting in normality. A linear mixed model with restricted maximum likelihood method was established based on a first-order autoregressive structure and used to analyse variables for main effects for time, group and sex, and all possible 2-way and 3-way interactions, with BMI and age included as random effects due to their inclusion as stratification variables. Differences between groups at baseline were analysed using independent *t* tests. Bivariate correlations using Pearson’s correlation co-efficient were calculated to assess relationships between variables and changes in LBM. All analyses were performed using SPSS version 25 (IBM Corp, Armonk, NY, 2017). A *p* < 0.05 was considered significant for all tests.

## Results

### Participant characteristics

There were a total of 17 completers in each group, with a nearly even split of males and females (IFT = 9 males and 8 females, CERT = 8 males and 9 females). Overall, 10 participants (*n* = 8 female and *n* = 2 male) failed to complete the interventions (CERT = 5, IFT = 5). Of these 10, 3 were unable to commit to the exercise sessions, 2 were unable to commit to the dietary protocol (IFT = 1, CERT = 1), and the remaining 5 dropped out due to unrelated medical issues or relocation. Baseline characteristics for participants are presented in Table 2. No significant differences were found at baseline between groups as a whole, or when split by sex (Supplementary Table 1).

**Table 2:** Baseline participant characteristics

Baseline variables	IFT	CERT	*p* value^a^
	(*n* = 17; 9 males, 8 females)	(*n* = 17; 8 males, 9 females)	
Age (years)	24.7 (4.8)	23.2 (3.9)	0.31
Height (m)	1.72 (0.1)	1.71 (0.1)	0.82
Weight (kg)	80.1 (13.8)	79.6 (13.5)	0.92
BMI (kg/m^2^)	27.0 (2.7)	27.1 (2.9)	0.93
LBM (kg)	54.3 (12.7)	53.3 (13.1)	0.82
Body fat percentage (%)	35.8 (8.6)	36.7 (7.9)	0.76
Bench press 3RM (kg)	43.3 (18.5)	39.5 (19.5)	0.56
Bench press volume (70% 1RM) (kg)	456.6 (229.1)	376.6 (171.3)	0.26
Leg Press 3RM (kg)	112.1 (54.1)	108.1 (65.8)	0.85
Leg press volume (70% 1RM) (kg)	1223.6 (803.3)	999.1 (631.3)	0.37

Mean (*SD*)

*BMI* body mass index, *LBM* lean body mass, *1RM* 1 repetition maximum, *3RM*  3 repetition maximum

^a^*P* values reported are for independent *t* tests between intervention groups

### Bodyweight and body composition analysis

Bodyweight and body composition measured before and after the intervention are presented in Table 3. There was a main effect for time for weight, BMI, body fat mass, body fat percentage and LBM, with significant reductions in weight, BMI, body fat mass and body fat percentage observed in both dietary groups, whereas LBM was significantly increased in both groups. There was also a main effect for sex for weight, LBM, fat mass and body fat percentage, with lower weight and LBM, but higher fat mass and body fat percentage noted for females. A significant time x sex interaction was evident for weight, BMI and LBM, with larger reductions in weight and BMI observed in males, but greater increases in LBM observed in females. Individual changes in weight, LBM and body fat can be seen in Supplementary Fig. 4.

**Table 3:** The effects of 12 weeks of IFT and CERT with resistance training on body weight, BMI, body composition, pQCT and ultrasound variables in male and female participants

	Group	Baseline	Week 12	Δ (95% CI)	Δ % (95% CI)	*P* (group)	*P* (time)	*P* (sex)	*P* (I)	*P* (S)
Body composition variables										
BMI (kg/m^2^)	IFT Males (*n* = 9)	26.6 (3.0)	25.1 (2.7)	− 1.5 (− 2.1, − 0.9)	− 5.6 (− 7.9, − 3.4)	0.98	< 0.001*	0.62	0.64	0.007****
	CERT Males (*n* = 8)	27.6 (2.4)	25.5 (2.7)	− 2.1 (− 2.5, − 1.5)	− 7.6 (− 9.1, − 5.4)					
	IFT Females (*n* = 8)	27.5 (2.5)	26.5 (2.5)	− 1.0 (− 1.8, 0.0)	− 3.6 (− 6.5, 0.0)					
	CERT Females (*n* = 9)	26.7 (3.4)	26.0 (2.9)	− 0.7 (− 1.6, 0.2)	− 2.6 (− 6.0, 0.7)					
Weight (kg)	IFT Males (*n* = 9)	87.3 (12.5)	82.6 (11.7)	− 4.7 (− 6.7, − 2.8)	− 5.4 (− 7.7, − 3.2)	0.97	< 0.001*	< 0.001**	0.55	0.001****
	CERT Males (*n* = 8)	88.0 (11.5)	81.7 (12.1)	− 6.3 (− 7.7, − 4.9)	− 7.2 (− 8.8, − 5.6)					
	IFT Females (*n* = 8)	71.9 (10.7)	69.5 (9.8)	− 2.4 (− 5.0, 0.1)	– 3.3 (− 7.0, 0.1)					
	CERT Females (*n* = 9)	72.1 (10.6)	70.1 (8.8)	− 2.0 (− 4.4, 0.4)	− 2.8 (− 6.1, 0.6)					
LBM (kg)	IFT Males (*n* = 9)	64.4 (7.2)	65.7 (7.4)	1.3 (0.2, 2.4)	2.0 (0.3, 3.7)	0.99	< 0.001*	< 0.001**	0.47	0.003****
	CERT Males (*n* = 8)	64.6 (9.4)	65.0 (9.6)	0.4 (− 0.6, 1.5)	0.6 (− 0.9, 2.3)					
	IFT Females (*n* = 8)	43.0 (5.6)	45.4 (5.6)	2.4 (1.3, 3.7)	5.6 (3.0, 8.6)					
	CERT Females (*n* = 9)	43.2 (4.8)	45.8 (4.1)	2.6 (1.2, 4.0)	6.0 (2.8, 9.3)					
Fat mass (kg)	IFT Males (*n* = 9)	27.4 (8.9)	20.2 (6.3)	− 7.2 (− 10.3, − 4.1)	− 26.3 (− 37.6, − 15.0)	0.96	< 0.001*	0.002**	0.32	0.18
	CERT Males (*n* = 8)	28.2 (5.0)	19.3 (4.8)	− 8.9 (− 10.7, − 7.0)	− 31.6 (− 37.9, − 24.8)					
	IFT Females (*n* = 8)	32.8 (6.9)	26.0 (5.1)	− 6.8 (− 10.5, − 3.0)	− 20.7 (− 32.0, − 9.2)					
	CERT Females (*n* = 9)	32.3 (8.8)	26.2 (7.9)	− 6.1 (− 7.9, − 4.3)	− 18.9 (− 24.5, − 13.3)					
Body fat percentage (%)	IFT Males (*n* = 9)	29.4 (6.0)	23.1 (4.6)	− 6.3 (− 8.5, − 3.9)	− 21.4 (− 28.9, − 13.3)	0.95	< 0.001*	< 0.001**	0.64	0.75
	CERT Males (*n* = 8)	30.4 (3.7)	22.8 (4.3)	− 7.6 (− 9.2, − 6.0)	− 25.0 (− 30.3, − 19.7)					
	IFT Females (*n* = 8)	43.0 (3.9)	36.2 (2.2)	− 6.8 (− 9.7, − 4.0)	− 15.8 (− 22.6, − 9.3)					
	CERT Females (*n* = 9)	42.2 (6.3)	35.9 (6.7)	− 6.3 (− 7.9, − 4.8)	− 14.9 (− 18.7, − 11.4)					
pQCT variables										
Muscle (cm^2^)	IFT Males (*n* = 7)	172.5 (23.2)	170.8 (23.4)	− 1.7 (− 8.1, 4.7)	− 1.0 (− 4.7, 2.7)	0.40	0.10	< 0.001**	0.03***	0.21
	CERT Males (*n* = 8)	162.5 (17.7)	165.2 (18.2)	2.7 (− 1.1, 6.5)	1.7 (− 0.7, 4.0)					
	IFT Females (*n* = 8)	117.9 (21.3)	118.1 (17.8)	0.2 (− 6.3, 6.7)	0.2 (− 5.3, 5.7)					
	CERT Females (*n* = 8)	111.3 (12.2)	118.1 (8.1)	6.8 (1.7, 11.9)	6.1 (1.5, 10.7)					
Intra-muscular fat (cm^2^)^a^	IFT Males (*n* = 7)	1.03 (0.64)	0.68 (0.58)	− 0.35 (− 0.81, 0.13)	− 33.98 (− 78.64, 12.62)	0.61	0.01*	0.31	0.63	0.70
	CERT Males (*n* = 8)	2.30 (2.11)	1.33 (1.21)	− 0.97 (− 1.96, 0.02)	− 42.17 (− 0.87,					
	IFT Females (*n* = 8)	2.22 (1.08)	1.48 (0.78)	− 0.74 (− 1.86, 0.37)	− 33.33 (− 83.78, 16.67)					
	CERT Females (*n* = 8)	1.71 (1.00)	1.40 (0.71)	− 0.31 (− 0.98, 0.36)	− 18.13 (− 57.31, 21.05)					
Subcutaneous fat (cm^2^)	IFT Males (*n* = 7)	71.2 (10.5)	63.7 (18.3)	− 7.5 (− 20.3, 5.4)	− 10.5 (− 28.5, 7.6)	0.41	< 0.001^*^	< 0.001^**^	0.62	0.83
	CERT Males (*n* = 8)	79.7 (27.5)	64.9 (16.3)	− 14.8 (− 26.5, − 3.0)	− 18.6 (− 33.2, − 3.8)					
	IFT Females (*n* = 8)	134.8 (35.8)	116.3 (33.8)	− 18.5 (− 30.5, − 6.3)	− 13.7 (− 22.6, − 4.7)					
	CERT Females (*n* = 8)	142.6 (42.9)	136.6 (48.3)	− 6.0 (− 17.6, 5.5)	− 4.2 (− 12.3, 3.9)					
Ultrasound variables										
RF Thickness (cm)	IFT Males (*n* = 7)	1.83 (0.36)	1.98 (0.31)	0.15 (0.03, 0.25)	8.20 (1.64, 13.66)	0.73	< 0.001*	0.009**	0.05	0.09
	CERT Males (*n* = 5)	1.95 (0.27)	2.17 (0.28)	0.22 (0.11, 0.34)	11.28 (5.64, 17.43)					
	IFT Females (*n* = 6)	1.78 (0.15)	1.82 (0.24)	0.04 (− 0.13, 0.21)	2.25 (− 7.30, 11.80)					
	CERT Females (*n* = 9)	1.49 (0.26)	1.64 (0.27)	0.15 (0.09, 0.23)	10.07 (6.04, 15.44)					
RF CSA (cm^2^)	IFT Males (*n* = 7)	6.37 (1.09)	7.43 (1.37)	1.06 (0.33, 1.78)	16.64 (5.18, 27.94)	0.35	< 0.001*	0.002**	0.07	0.11
	CERT Males (*n* = 5)	6.85 (1.50)	7.96 (1.32)	1.11 (0.45, 1.76)	16.20 (6.57, 26.69)					
	IFT Females (*n* = 6)	6.16 (1.19)	6.19 (1.29)	0.03 (− 0.38, 0.43)	0.49 (− 6.17, 6.98)					
	CERT Females (*n* = 9)	4.28 (1.23)	5.14 (1.46)	0.86 (0.46, 1.27)	20.09 (− 10.75, 29.67)					
RF EI (arbitrary units)	IFT Males (*n* = 7)	22.1 (8.2)	15.9 (4.5)	− 6.2 (− 11.2, − 1.3)	− 28.1 (− 50.7, − 5.9)	0.43	< 0.001*	< 0.001**	0.47	0.95
	CERT Males (*n* = 5)	17.1 (6.6)	14.5 (3.7)	− 2.6 (− 7.0, 1.8)	− 15.2 (− 40.9, 10.5)					
	IFT Females (*n* = 6)	33.4 (14.0)	29.1 (15.0)	− 4.3 (− 7.4, − 1.3)	− 12.9 (− 22.2, − 3.9)					
	CERT Females (*n* = 9)	43.0 (10.6)	38.3 (11.3)	− 4.7 (− 10.4, 0.9)	− 10.9 (− 24.2, 2.1)					
VI Thickness (cm)	IFT Males (*n* = 7)	1.94 (0.37)	1.78 (0.20)	− 0.16 (− 0.48, 0.15)	− 8.25 (− 24.74, 7.73)	0.50	0.38	0.07	0.21	0.12
	CERT Males (*n* = 5)	1.63 (0.22)	1.55 (0.36)	− 0.08 (− 0.34, 0.17)	− 4.91 (− 20.86, 10.43)					
	IFT Females (*n* = 6)	1.47 (0.41)	1.42 (0.37)	− 0.05 (− 0.20, 0.10))	− 3.40 (− 13.61, 6.80)					
	CERT Females (*n* = 9)	1.49 (0.37)	1.62 (0.22)	0.13 (− 0.07, 0.33)	8.72 (− 4.70, 22.15)					
VI EI (arbitrary units)	IFT Males (*n* = 7)	38.4 (21.0)	35.6 (18.5)	− 2.8 (− 12.7, 7.0)	− 7.3 (− 33.1, 18.2)	0.99	0.75	0.17	0.65	0.90
	CERT Males (*n* = 5)	33.5 (16.1)	35.1 (7.2)	1.6 (− 20.6, 23.9)	4.8 (− 61.5, 71.3)					
	IFT Females (*n* = 6)	40.4 (10.5)	44.2 (29.2)	3.8 (− 21.6, 29.1)	9.4 (− 53.5, 72.0)					
	CERT Females (n = 9)	48.4 (10.9)	41.8 (9.1)	− 6.6 (− 15.4, 2.3)	− 13.6 (− 31.8, 4.8)					

Mean (*SD*)

*BMI* body mass index, *CSA* cross-sectional area, *EI* echogenicity, group = main effect for diet group, *I*  time × group interaction, *LBM* lean body mass, *RF* rectus femoris, *S* time × sex interaction, sex = main effect for sex, time = main effect for time, *VI* vastus intermedius. pQCT was conducted on *n* = 31. Ultrasound was conducted on *n* = 27

*Significantly different than baseline at week 12 in all groups combined

**Significantly different between males and females

***Significant time x group interaction

****Significant time x sex interaction

^a^Data reported are untransformed; statistical analyses for intra-muscular fat are based on Log10 transformed values

### Mid-thigh muscle surface area and intramuscular and subcutaneous fat analysis

#### pQCT

Muscle surface area and intramuscular and subcutaneous fat measured before and after the intervention via pQCT are reported in Table 3. A main effect for time was found for subcutaneous fat and log10 intramuscular fat with significant reductions occurring in both groups over time. A main effect for sex was also found for muscle surface area and subcutaneous fat, with larger muscle surface area, but lower subcutaneous fat noted for males. There was a time x group effect for muscle surface area, with those in the CERT group experiencing a mean increase in muscle surface area compared to the IFT group.

#### Ultrasound

RF thickness, CSA and EI, and VI thickness and EI measured before and after the intervention via ultrasound are presented in Table 3. A main effect for time for RF thickness, RF CSA and RF EI was noted, with RF thickness and CSA significantly increased in both groups over time, whereas RF EI significantly decreased in both groups over time. A main effect for sex was also found for RF thickness, RF CSA and RF EI, with larger RF thickness and CSA, but lower RF EI in males observed. There were no other significant interactions or main effects identified for RF measurements and/or all VI assessments.

### Dietary intake analysis

Participant dietary intake data measured before and during the intervention are summarised in Table 4. There was a main effect for time for overall mean absolute daily energy intake in kJ, relative energy intake in kJ/kg, relative protein intake in g/kg, relative carbohydrate intake in g/kg and relative fat in g/kg, with significant reductions in energy (both absolute and relative), relative carbohydrate and fat intake identified across all groups, and a significant increase in relative protein intake. There was also a main effect for sex for mean absolute daily energy intake in kJ, with lower consumption in females. A significant time x sex interaction was found for relative energy intake (kJ/kg) and relative carbohydrate intake (g/kg), with females demonstrating greater reductions in energy and carbohydrate intake compared to males during the intervention period. Average energy restriction for those in the IFT and CERT groups were 31.0 ± 5.9% and 29.3 ± 6.5% for males and 33.9 ± 7.1% and 28.1 ± 6.1% for females respectively, with no significant difference between groups overall (IFT = 32.4 ± 6.4% versus CERT = 28.7 ± 6.1%).

**Table 4:** The effects of 12 weeks of IFT and CERT with resistance training on energy and macronutrient intake in male and female participants

Diet variable	Group	Baseline	During Intervention^a^	Δ (95% CI)	Δ % (95% CI)	*P* (group)	*P* (time)	*P *(sex)	*P* (I)	*P* (S)
Mean daily energy intake (kJ)	IFT males (*n* = 9)	8134 (1993)	7678 (909)	− 456 (− 1892, 980)	− 5.6 (− 23.3, 12.0)	0.32	0.006*	0.006**	0.69	0.09
	CERT males (*n* = 8)	8222 (1930)	7981 (836)	− 241 (− 1951, 1467)	− 2.9 (− 23.7, 17.8)					
	IFT females (*n* = 8)	7041 (957)	5538 (646)	− 1503 (− 2325, − 680)	− 21.3 (− 33.0, − 9.7)					
	CERT females (*n* = 9)	7469 (1939)	6219 (680)	− 1250 (− 2533, 33)	− 16.7 (− 33.9, 0.4)					
Energy (kJ/kg)	IFT males (*n* = 9)	94 (25)	91 (12)	− 3 (− 20, 13)	− 3.2 (− 21.3, 13.8)	0.43	0.008*	0.91	0.69	0.03***
	CERT males (*n* = 8)	95 (27)	94 (10)	− 1 (− 20, 18)	− 1.1 (− 21.1, 19.0)					
	IFT females (*n* = 8)	100 (21)	79 (9)	− 21 (− 36, − 7)	− 21.0 (− 36.0, − 7.0)					
	CERT females (*n* = 9)	106 (32)	88 (13)	− 18 (− 37, 2)	− 17.0 (− 34.9, 1.9)					
Protein (g/kg)	IFT males (*n* = 9)	1.28 (0.52)	1.50 (0.22)	0.22 (− 0.15, 0.59)	17.19 (− 11.72, 46.10)	0.80	< 0.001*	0.26	0.48	0.46
	CERT males (*n* = 8)	1.08 (0.33)	1.53 (0.27)	0.45 (0.17, 0.73)	41.67 (15.74, 67.59)					
	IFT females (*n* = 8)	1.05 (0.15)	1.31 (0.13)	0.26 (0.13, 0.40)	24.76 (12.38, 38.10)					
	CERT females (*n* = 9)	1.18 (0.38)	1.42 (0.15)	0.24 (0.01, 0.50)	20.34 (0.85, 42.37)					
Carbohydrate (g/kg)	IFT males (*n* = 9)	2.22 (0.85)	2.07 (0.59)	− 0.16 (− 0.65, 0.34)	− 6.33 (− 29.28, 15.32)	0.31	0.001*	0.66	0.51	0.01***
	CERT males (*n* = 8)	2.36 (0.71)	2.28 (0.42)	− 0.09 (− 0.46, 0.29)	− 3.39 (− 19.49, 12.29)					
	IFT females (*n* = 8)	2.46 (0.92)	1.61 (0.39)	− 0.85 (− 1.50, − 0.19)	− 34.55 (− 60.98, − 7.72)					
	CERT females (*n* = 9)	2.56 (0.66)	1.94 (0.36)	− 0.62 (− 1.11, − 0.12)	− 24.22 (− 43.36, − 4.69)					
Fat (g/kg)	IFT males (*n* = 9)	0.87 (0.34)	0.76 (0.16)	− 0.11 (− 0.33, 0.11)	− 12.64 (− 37.93, 12.64)	0.61	< 0.001*	0.27	0.56	0.34
	CERT males (*n* = 8)	0.93 (0.33)	0.73 (0.16)	− 0.20 (− 0.47, 0.07)	− 18.68 (− 50.54, 7.53)					
	IFT females (*n* = 8)	1.05 (0.24)	0.73 (0.07)	− 0.32 (− 0.48, − 0.13)	− 30.48 (− 45.71, − 12.38)					
	CERT females (*n* = 9)	0.99 (0.33)	0.79 (0.17)	− 0.20 (− 0.39, − 0.03)	− 20.20 (− 39.39, − 3.03)					

Mean (*SD*)

Group = main effect for diet group, *I* = time x group interaction, *S* = time x sex interaction, sex = main effect for sex, time = main effect for time

*Significantly different than baseline at week 12 in all groups combined

**Significantly different between males and females

***Significant time x sex interaction

^a^Mean of week 1, 6 and 12 intakes

#### Fasting versus non-fasting days in IFT participants

Differences in dietary energy and protein intake on fasting and non-fasting days for the IFT group are presented in Table 5. Energy intake on fasting days for females was significantly lower in week 12 compared to week 1. No other significant differences between time points on fasting or non-fasting days were found.

**Table 5:** Dietary intake for fasting and non-fasting days for IFT male and female participants

Diet variable	Time	IFT males non-fast days	IFT males fast days	IFT females non-fast days	IFT females fast days
		(*n* = 9)	(*n* = 9)	(*n* = 8)	(*n* = 8)
Mean daily energy intake (kJ)	Week 1	9596 (1028)	2456 (136)	7185 (1041)	1957 (344)
	Week 6	9682 (1426)	2502 (431)	6758 (856)	1951 (260)
	Week 12	9380 (1712)	2511 (0)	6530 (1075)	1686 (292)^*^
	Overall mean^a^	9619 (1058)	2490 (175)	6824 (734)	1865 (276)
Energy (kJ/kg)	Week 1	113 (14)	29 (4)	100 (19)	27 (4)
	Week 6	116 (22)	30 (5)	97 (16)	28 (4)
	Week 12	114 (24)	31 (4)	94 (14)	24 (5)
	Overall mean^a^	114 (17)	29.62 (4.03)	97 (13)	26 (4)
Protein (g/kg)	Week 1	1.62 (0.40)	1.07 (0.15)	1.54 (0.19)	0.98 (0.12)
	Week 6	1.70 (0.39)	1.08 (0.21)	1.45 (0.28)	0.99 (0.16)
	Week 12	1.68 (0.41)	1.14 (0.16)	1.30 (0.21)	0.94 (0.17)
	Overall mean^a^	1.63 (0.35)	1.11 (0.15)	1.44 (0.16)	0.98 (0.13)

Mean (*SD*)

*Significantly different to week 1 values in specified group, *p* < 0.05

^a^Mean of week 1, 6 and 12 intakes

### Upper body and lower body 3RM strength and endurance volume analysis

Changes in the bench press and leg press 3RM, and bench press and leg press endurance test volume are reported in Table 6. There were main effects for time for bench press 3RM, bench press volume, leg press 3RM and leg press volume, with increases noted in each of these variables across the intervention. There were also main effects for sex for bench press and leg press 3RM, and bench press and leg press volume, with lower values reported in females. No other significant interactions or main effects were identified for strength or endurance variables.

**Table 6:** The effects of 12 weeks of IFT and CERT with strength training on strength variables in male and female participants

Strength variable	Group	Baseline	Week 12	Δ (95% CI)	Δ % (95% CI)	*P* (group)	*P* (time)	*P* (sex)	*P* (I)	*P* (S)
Bench press 3RM (kg)	IFT Males (*n* = 9)	58.0 (12.1)	61.7 (11.3)	3.7 (− 0.1, 7.5)	6.4 (− 0.2, 12.9)	0.67	< 0.001*	< 0.001**	0.35	0.07
	CERT Males (*n* = 8)	57.0 (12.9)	61.1 (13.9)	4.1 (1.7, 6.5)	7.2 (3.0, 11.4)					
	IFT Females (*n* = 8)	26.8 (4.9)	31.9 (3.0)	5.1 (2.9, 7.5)	19.0 (10.8, 28.0)					
	CERT Females (*n* = 9)	23.9 (30.8)	30.8 (5.2)	6.9 (5.5, 8.3)	28.9 (23.0, 34.7)					
Bench press volume (70% 1RM) (kg)	IFT Males (*n* = 9)	611 (197)	692 (190)	81 (− 12, 174)	13.3 (− 2.0, 28.5)	0.29	< 0.001*	< 0.001**	0.29	0.46
	CERT Males (*n* = 8)	522 (128)	657 (103)	135 (53, 216)	25.9 (10.2, 41.4)					
	IFT Females (*n* = 8)	283 (104)	406 (61)	123 (49, 196)	43.5 (17.3, 69.3)					
	CERT Females (*n* = 9)	247 (66)	394 (64)	147 (67, 226)	59.5 (27.1, 91.5)					
Leg Press 3RM (kg)	IFT Males (*n* = 9)	150.6 (37.1)	185.0 (44)	34.4 (11.3, 57.6)	22.8 (7.5, 38.2)	0.95	< 0.001*	< 0.001**	0.51	0.34
	CERT Males (*n* = 8)	165.0 (47.9)	186.8 (48)	21.8 (11.4, 32.3)	13.2 (6.9, 19.6)					
	IFT Females (*n* = 8)	68.8 (32.8)	101.8 (33)	33.0 (18.3, 48.0)	48.0 (26.6, 69.8)					
	CERT Females (*n* = 9)	57.4 (22.8)	93.9 (22)	36.5 (27.0, 45.9)	63.6 (47.0, 80.0)					
Leg press volume (70% 1RM) (kg)	IFT Males (*n* = 9)	1636 (782)	2356 (1154)	720 (123, 1315)	44.0 (7.5, 80.4)	0.36	< 0.001*	< 0.001**	0.74	0.95
	CERT Males (*n* = 8)	1468 (578)	1904 (345)	436 (139, 732)	29.7 (9.5, 49.9)					
	IFT Females (*n* = 8)	759 (558)	1269 (840)	510 (154, 866)	67.2 (20.3, 114.1)					
	CERT Females (*n* = 9)	582 (1248)	1248 (526)	666 (299, 1033)	114.4 (51.4, 177.5)					

Mean (*SD*)

Group = main effect for diet group, *I* = time x group interaction, *S* = time x sex interaction, sex = main effect for sex, time = main effect for time, *1RM* 1 repetition maximum, *3RM* 3 repetition maximum

*Significantly different than baseline at week 12 in all groups combined

**Significantly different between males and females

### Correlations between variables and changes in LBM

**Fig. 2:**
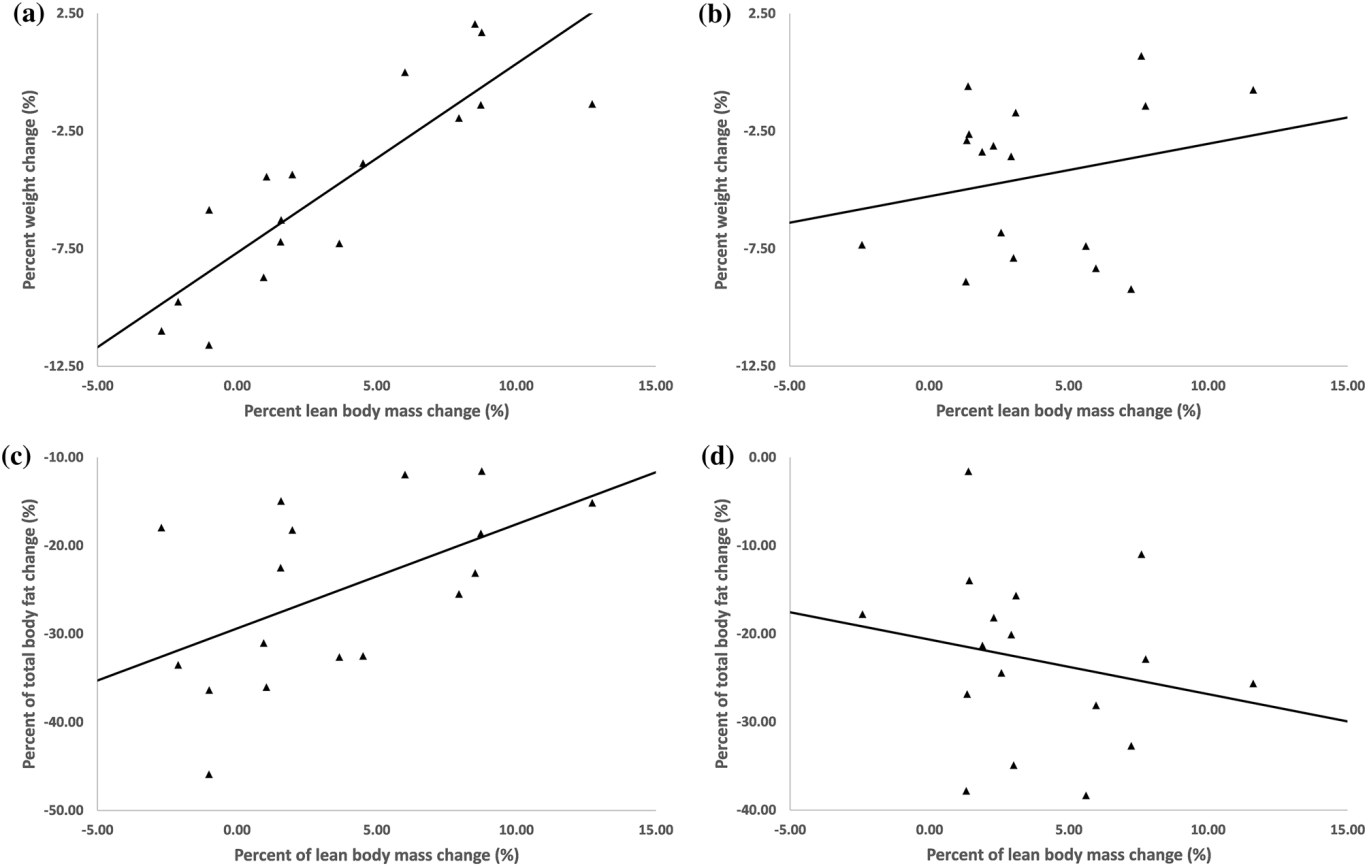
Bivariate correlations between changes in body weight and LBM in CERT (**a**) and IFT (**b**) groups; and between changes in percentage of total fat mass lost and LBM in CERT (**c**) and IFT (**d**) groups

Figure 2 shows the correlation between relative changes in body weight and body fat and LBM. There was a significant, moderate positive correlation between changes in LBM and weight in both groups overall (*r* = 0.63, *p* < 0.001), indicating that as weight loss increased, gains in LBM reduced. When split by intervention group, this relationship was strengthened in the CERT group (*r* = 0.86, *p* < 0.001), while no significant relationship was seen for the IFT group (*r* = 0.23, *p* = 0.38), with all but one IFT group participant increasing LBM regardless of percentage weight loss. Similarly, there was a moderate, significant correlation between the percentage of overall fat mass lost with changes in LBM in the CERT group (*r* = 0.53, *p* = 0.03), but not in the IFT group (*r* = − 0.21, *p* = 0.41). Change in LBM was negatively correlated with absolute relative energy (kJ/kg) (*r* = − 0.40, *p* = 0.02) and carbohydrate (g/kg) intake (*r* = − 0.36, *p* = 0.03) during the intervention and positively correlated with percent change in pQCT muscle area (*r* = 0.51, *p* = 0.003). Relative change (percent) in bench press 3RM (*r* = 0.48, *p* = 0.004) and leg press 3RM (*r* = 0.55, *p* = 0.001) were also positively correlated with changes in LBM. No other measurements were found to have significant correlations with change in LBM.

## Discussion

To the authors’ knowledge, this is the first randomised trial to have compared the effects of 5:2 intermittent fasting and continuous energy restriction on body composition and strength adaptations when concurrently undertaking resistance training. Our findings suggest that when overall dietary energy and protein intake are similar, both energy-restricted diets induced comparable increases in LBM and strength, and reductions in weight and fat when combined with a 12 week resistance training program. In contrast, some differences were noted between groups in local assessments of the thigh muscle. Sex-dependent differences were also observed in both groups, specifically in weight and LBM responses, with males demonstrating greater weight loss, but less gains in LBM. This could be a reflection of weight-loss-induced impairment of LBM accrual, however; this concept requires further exploration.

In the current study, both dietary intervention groups experienced significant reductions in body fat (mean − 7.2 kg; 95% CI − 8.4 kg, − 6.0 kg) and increases in LBM (mean + 1.7 kg; 95% CI + 1.1 kg, + 2.3 kg) suggesting weight lost was exclusively from fat. While whole-body composition changes were similar between groups, increases in LBM have previously been more commonly reported in studies of individuals undertaking resistance training with continuous energy restriction than with intermittent fasting [39–43]. This could be a reflection of differences in daily energy restriction amounts but also study design, especially the training program. Studies demonstrating increases in LBM while utilising continuous energy restriction have commonly employed more frequent and/or intense training protocols (i.e. 5–7 days/week) compared to the common 3 days/week model typically used in intermittent fasting studies [16] and the present study. However, a 3 day/week training program could have important practical implications given it may be more appealing/realistic to undertake for a significant proportion of the general population. To our knowledge, only one other study has demonstrated significant increases in LBM when intermittent fasting and resistance training were combined [19]. In this study, per-protocol analysis showed an increase of 1.4 and 1.0 kg in LBM in trained women undertaking resistance training with and without β-hydroxy β-methylbutyrate (HMB), respectively. However, as no concomitant changes in weight were observed, it is likely that participants were in energy balance. This could explain why these participants gained LBM, in contrast to other studies which have demonstrated small to moderate reductions in weight (− 1.0 kg to − 3.3 kg) alongside small losses, no change, or statistically non-significant increases in LBM (− 0.4 kg to + 0.6 kg) [17, 18, 20, 39]. The paucity of studies investigating intermittent fasting with resistance training makes it difficult to ascertain if LBM can be gained when both are combined in conjunction with a concomitant energy deficit [16]. Moreover, intermittent fasting is a broad concept, and while alternate day fasting, time-restricted feeding and 5:2 all fit under this umbrella, there are significant differences in how these diets are applied. For example, while time-restricted feeding uses extended periods of fasting each day (16–20 h), if an energy deficit is prescribed, it is generally on a daily basis. On the other hand, while alternate day fasting and 5:2 IF are similar in that they generally prescribe severe restrictions on fasting days and no restrictions on non-fasting days, the number of fasting days each week is different. Therefore, it is difficult to make direct comparisons between these results and those of previous studies, even if the diets used are all broadly classified as intermittent fasting.

Ultrasound assessment showed a non-significantly (*p* = 0.05) greater increase in RF thickness in the CERT compared to the IFT group (CERT =  + 0.18 cm; 95% CI + 0.13 cm, + 0.24 cm versus IFT =  + 0.10 cm; 95% CI + 0.01 cm, + 0.19 cm), with similar non-significant (*p* = 0.07) findings for RF CSA (CERT =  + 0.95 cm^2^; 95% CI + 0.65 cm^2^, + 1.25 cm^2^ versus IFT =  + 0.58 cm^2^; 95% CI + 0.09 cm^2^, + 1.07 cm^2^). Similarly, pQCT demonstrated significantly (*p* = 0.03) greater increases in muscle area in the CERT group compared to IFT group (CERT =  + 4.75 cm^2^; 95% CI + 1.76 cm^2^, + 7.73 cm^2^ versus IFT = − 0.69 cm^2^; 95% CI − 4.67 cm^2^, + 3.29 cm^2^). Observed differences between groups at the local musculature level could be due to a number of reasons. Firstly, it is known that muscle hypertrophy in response to resistance training does not occur uniformly throughout the body, with a upper body more responsive than lower body muscle mass [40–42] and thus it is likely that we missed changes in the upper body musculature by only assessing the thigh muscle. In support of this, it was noted that when examining DXA-derived regional differences in LBM, the IFT group showed greater relative increases in the upper body compared to those in the CERT group, albeit marginally, while the opposite was true for the lower body (Supplementary Table 2). Secondly, our small, uneven sample size may have been insufficient to identify real differences between diet groups when utilising more sensitive assessments of muscle, especially given the known large inter-individual variability in adaptations to resistance training [43]. Despite the differences, change in muscle surface area as measured by pQCT was positively correlated with changes in whole-body LBM (*r* = 0.51) for both groups combined, suggesting muscular hypertrophy may have underpinned the increases in LBM. These findings support the notion that muscle accrual can occur together with reductions in fat mass when energy deficits are combined with resistance training and protein intakes above standard daily recommendations [44–48].

Notable differences between sexes in terms of the magnitude of LBM change were evident in the present study. Female participants gained significantly more LBM on average (+ 2.5 kg; 95% CI + 1.7 kg, + 3.3 kg versus + 0.9 kg; 95% CI + 0.2 kg, + 1.6 kg) compared to males, but experienced less weight loss (− 2.2 kg; 95% CI − 3.8 kg, − 0.7 kg versus − 5.5 kg; 95% CI − 6.7 kg, − 4.4 kg). A number of sex-specific factors may explain the disparity in weight and fat reduction between males and females. First, although all participants were prescribed similar relative energy deficits (20–23%—with slight variation due to the standardised nature of fasting days), comparatively larger lean mass in males (and therefore higher energy requirements) would have led to a greater absolute energy deficit for males by ~ 600 kJ/day. Given that the reported energy deficit for both sexes was ~ 10% more than prescribed (~ 30%), the absolute difference between sexes was most likely greater than this. When combined with the fact that females may require a greater absolute energy deficit per unit of weight-loss [49], are more likely to under-report energy intake [50] and may be impacted by hormones that promote weight retention [51, 52], it is not surprising that males experienced greater weight and fat loss in the current study. Additionally, differences in total amounts (and therefore overall rate) of weight loss between sexes could partially explain the greater LBM gains in females compared to males. Previous research has shown that slower rates of weight loss may be beneficial for LBM preservation, though this finding is not ubiquitous in the literature [53]. Congruent with this, when all weight loss and LBM changes for both sexes were pooled together in the current study, there was a moderate, positive correlation between change in weight and change in LBM (*r* = 0.63). That is, as weight loss increased, LBM gains were reduced and potentially compromised. However, when split by diet, this relationship strengthened in the CERT group (*r* = 0.86), but weakened in the IFT group to be statistically non-significant (*r* = 0.23). This trend was also seen when comparing a relative overall fat loss with changes in LBM. These findings may suggest that when paired with resistance training, the 5:2 style diet could be protective/promote gains in LBM compared with continuous energy restriction when greater amounts of weight loss occur. However, given the contrasting observations in pQCT and ultrasound assessments, which favoured the CERT group in some measurements, this concept is speculative and requires further investigation.

Upper and lower body muscle strength and endurance were significantly increased over the 12 week period, regardless of dietary intervention. There was a moderate, positive correlation between increases in LBM and 3RM for both bench press (*r* = 0.48) and leg press (*r* = 0.55), suggesting that some of this increase may be attributable to growth in LBM, although given the untrained population studied, it is probable that neuromuscular adaptations also contributed significantly [42]. Regardless, this provides evidence that when combined with resistance training, both diets appeared to support increases in strength in a comparable manner.

There were a number of strengths of the current study. First, participants were supervised for the majority of their training sessions, allowing standardisation of form and effort. Second, frequent access to the study dietitian may have helped improve motivation and compliance. Third, we utilised non-invasive and reliable methods of assessment to measure changes in muscle size and quality alongside whole body LBM (pQCT and ultrasound), both of which have shown utility in clinical settings [54, 55].

Our study was also subject to a number of limitations. First, while frequent supervision and feedback for training and diet may have helped with study validity, this may not be practical for the general population. Second, although we collected detailed dietary data, the limitations of self-reported dietary intake are well known [56], and our ability to make strong inferences based on dietary data were limited. However, reported intake did reflect the aims of our study (i.e. increased intake on non-fasting days for the IFT compared to the CERT group). Third, it is unclear how the provision of supplements to the IFT group on fasting days may have affected compliance, or whether the supplements themselves may have contributed to the results. Fourth, as we did not record menstrual cycle for females, it is unclear whether this may have affected our results. Finally, the sample size calculation used in the present study was based on research using similar population demographics and study designs available at the time [16, 23, 38]. However, the effect size determined by the magnitude of difference in the primary outcome measure (i.e. LBM) does appear to be larger than those observed in more contemporary studies that focus on intermittent fasting and resistance training. Thus, the final sample size in the current study may have been too low to detect smaller (but potentially clinical meaningful) differences in LBM that could arise from similar interventions, and future research may benefit from including greater numbers to overcome this limitation.

In summary, 5:2 intermittent fasting or continuous energy restriction combined with moderate protein intake and resistance training over 12 weeks led to comparable LBM gains, weight and body fat loss, and improvements in muscle strength, endurance and quality. However, there were clear sexual dimorphic differences in a number of these outcomes, and some disparities between groups when the measurement of whole-body changes were compared to localised muscle changes. Future studies should investigate the longer-term impacts of 5:2 fasting in comparison to continuous energy restriction, but importantly, explore the relationship between transient changes in energy balance and protein turnover, weight loss and muscle mass accrual.

## Supplementary Information

Below is the link to the electronic supplementary material.

Supplementary file1 (PDF 514 KB)

## Abbreviations

CSA: Cross-sectional area
CERT: Continuous energy restriction plus training group
DXA: Dual x-ray absorptiometry
EI: Echogenicity
IFT: Intermittent fasting plus training group
LBM: Lean body mass
MPB: Muscle protein breakdown
MPS: Muscle protein synthesis
pQCT: Peripheral quantitative computed tomography
RF: Rectus femoris
VI: Vastus intermedius
1RM: 1-Repetition maximum
3RM: 3-Repetition maximum
