# Supplementary material for: Intermittent fasting and continuous energy restriction result in similar changes in body composition and muscle strength when combined with a 12 week resistance training program
Source: Eur J Nutr. 2022 Jan 27;61(4):2183–99. doi: 10.1007/s00394-022-02804-3 (PMC9106626; doi:10.1007/s00394-022-02804-3)
Supplement: Supplementary file 1 — Supplementary file1 (PDF 514 KB) [file 394_2022_2804_MOESM1_ESM.pdf]

Supplementary material 1. Example exercise program, ultrasound and pQCT analysis, individual body composition changes, baseline characteristics compared by sex, regional body composition differences.

Intermittent fasting and continuous energy restriction result in similar changes in body composition and muscle strength when combined with a 12 week resistance training program

Authors

Stephen J. Keenan[1], Matthew B. Cooke[1,2], Ebrahim Bani Hassan[2,3], Won Sun Chen[1], Josef Sullivan[1], Sam X. Wu[1], Doa El-Ansary[1,4], Mahdi Imani[2,3], Regina Belski[1]

1. Faculty of Health, Arts and Design, School of Health Sciences, Swinburne University of Technology, Melbourne, Australia.
2. Australian Institute for Musculoskeletal Science (AIMSS), The University of Melbourne, VIC, Australia.
3. Department of Medicine, Western Health, Melbourne Medical School, The University of Melbourne, VIC, Australia.
4. Department of Surgery, Melbourne Medical School, The University of Melbourne, VIC, Australia

Corresponding author: Stephen J. Keenan – skeenan@swin.edu.au

Supplemental Figure 1.

Example of a Full body workout

Warmup

5-10 minute warm up – stretching, jogging on the spot.

Workout

Supersets (exercise 1 superset with exercise 2 in each exercise pair). Continue until they can complete 3 sets of 15 reps of each exercise (aim to hit minimum of 12 repetitions), then progress. 2 minutes rest between sets.

Superset 1

Exercise 1

Push ups

Progressions:

1. Wall push ups (Keep distance from wall consistent/standard with all participants)
2. Knee push ups
3. Standard push ups
4. Depth push up (performed on weights)
5. Elevated push up (legs elevated)
6. Elevated push ups weighted (plate on back)

Exercise 2

Squats

Progressions:

1. Bodyweight parallel squat
2. Bodyweight full squat
3. Goblet squat (with dumbbell) parallel
4. Single leg movement (e.g Bulgarian split squat) with or without dumbbells (for example, if they can do single leg and achieve 15 repetitions easily, then add dumbbells so that they can just reach 12 repetitions, continue to progress through heavier dumbbells)

Superset 2

Exercise 1

Bent over rows

Progressions:

1. Resistance bands
2. Dumbbells – progression to heavier dumbbells
3. Dumbbells with resistance bands (or cable rows with increasing weight)

Exercise 2

Lunges

Progressions:

1. Static lunge (with or without weight)
2. Forward lunge
3. Forward lunge with weight (2 dumbbells or weight plate and progress heavier)
4. Jump split squat

Superset 3

Exercise 1

Bicep curls

Progressions:

1. Resistance band curls
2. Dumbbell curls – progress to heavier dumbbells

Exercise 2

Dips

Progressions:

1. Bent knee chair/bench dips
2. Straight leg chair/bench dips
3. One leg raised chair/bench dips
4. Both legs elevated chair/bench tips
5. Dumbbell on lap elevated leg chair/bench dips – progress to heavier dumbbells

Home workout

Progressions made by increasing time on each exercise by 5 seconds each week when time is completed with good form. 2 minutes rest between each superset, 3 sets of each superset.

Superset 1

30 seconds plank

30 seconds mountain climbers

Superset 2

30 seconds crunches/sit ups

30 seconds burpees

Superset 3

30 seconds side toe touches

30 seconds hip bridge

Cool down

Supplemental Figure 2. Sample ultrasound images of the anterior thigh muscle group (quadriceps); muscle morphology: thickness and cross-sectional area, and muscle composition; echogenicity

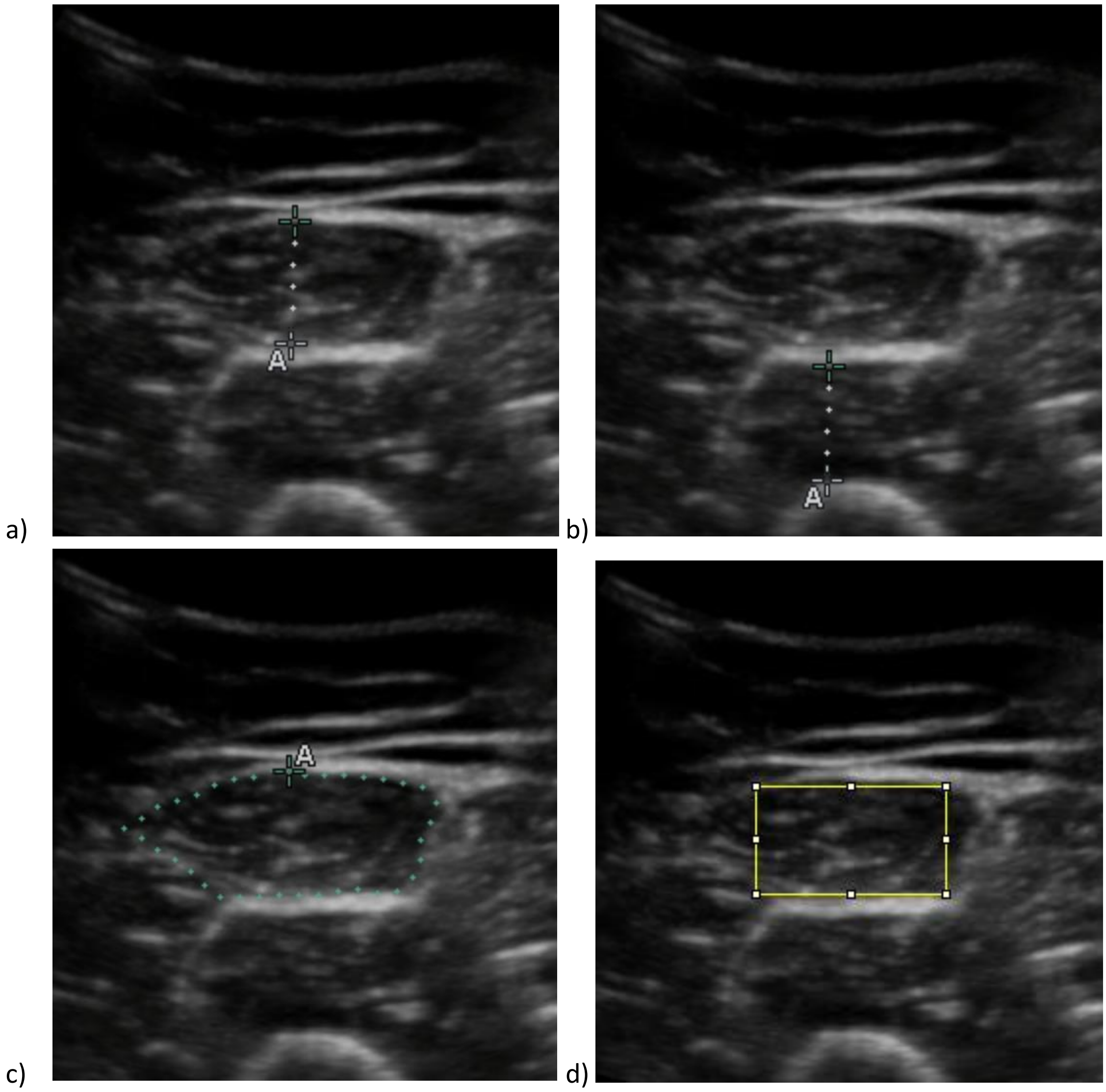

Key: Each image is oriented anterior to posterior and demonstrate how the measures were conducted: a) rectus femoris thickness; b) vastus intermedius thickness; c) rectus femoris cross-sectional area and; d) rectus femoris echogenicity.

Supplemental Figure 3. Example of pQCT analysis.

a) 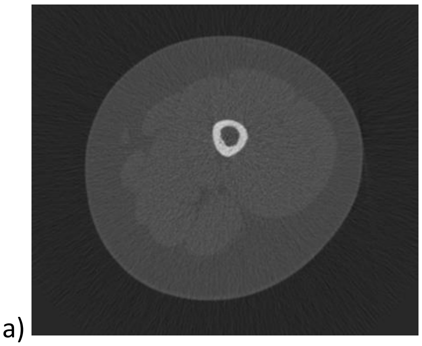

b) 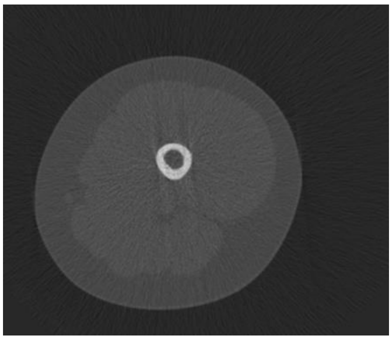

c) 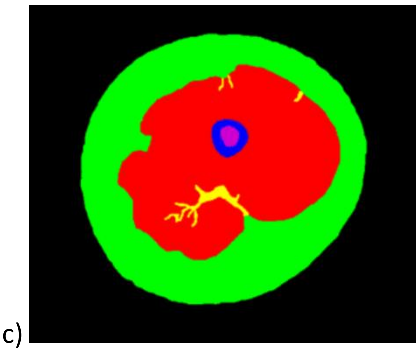

d) 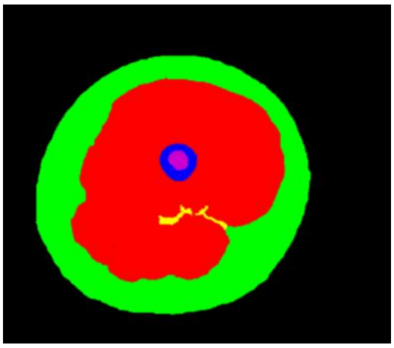

Key: a) and b) show unanalysed pQCT image of the mid-thigh of a subject pre- and post-intervention, respectively. c) and d) present segmented (quantified) a) and b) images, respectively. Note the significant decline of subcutaneous and intramuscular fat volume and, and increased muscle cross-sectional area post-intervention.

Green = subcutaneous fat; yellow = intramuscular adipose tissue; red = muscle and; blue = bone.

Supplemental figure 4. Individual changes in weight, lean body mass and body fat.

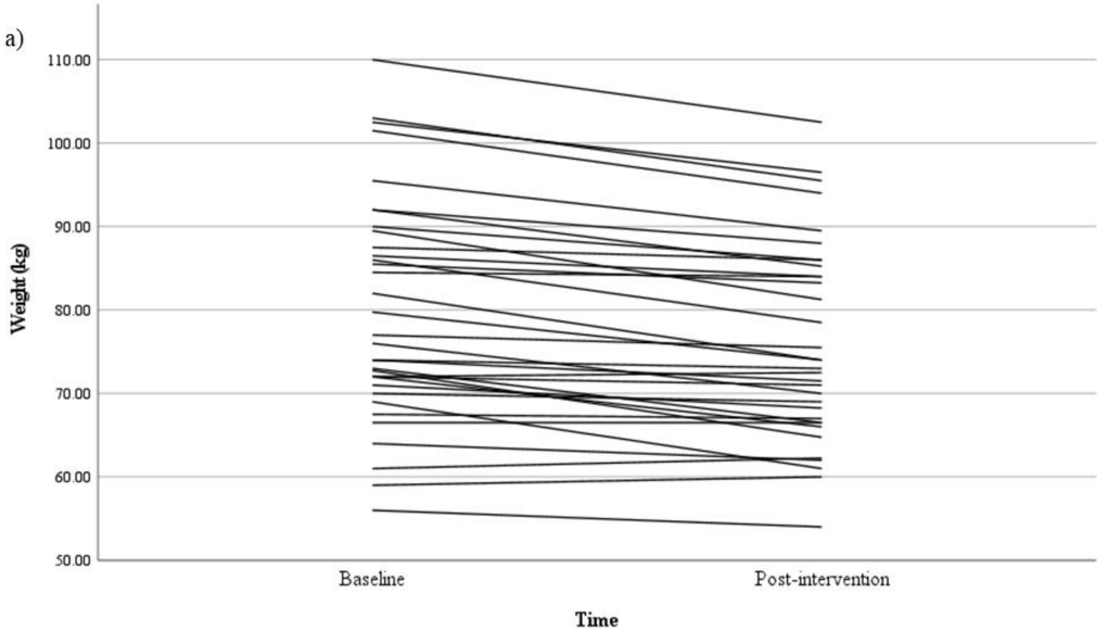

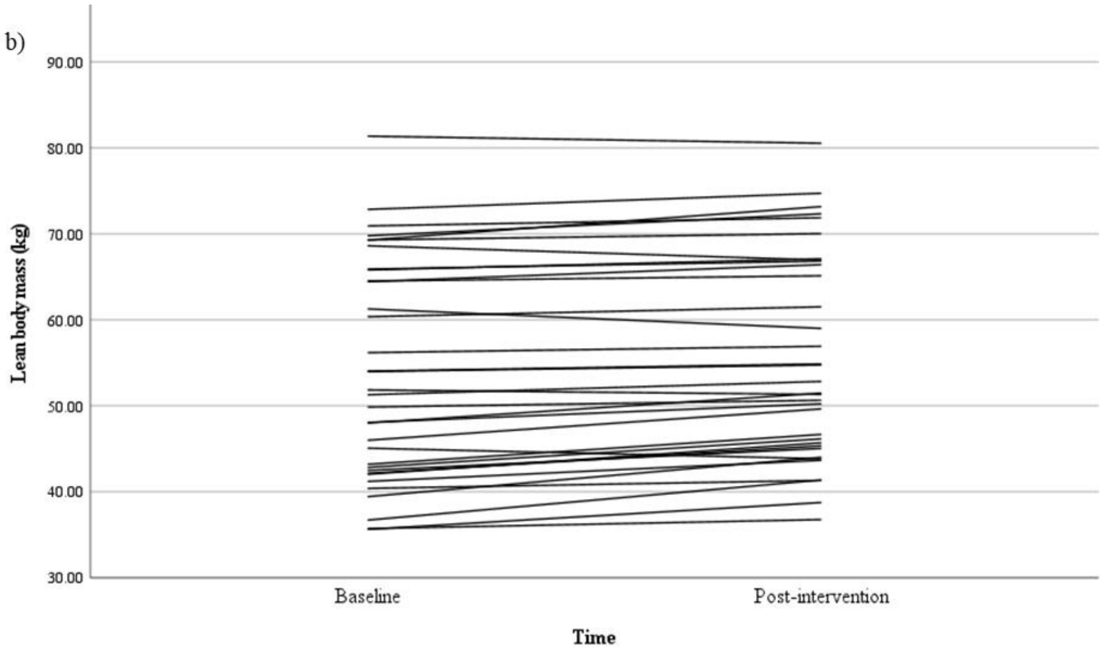

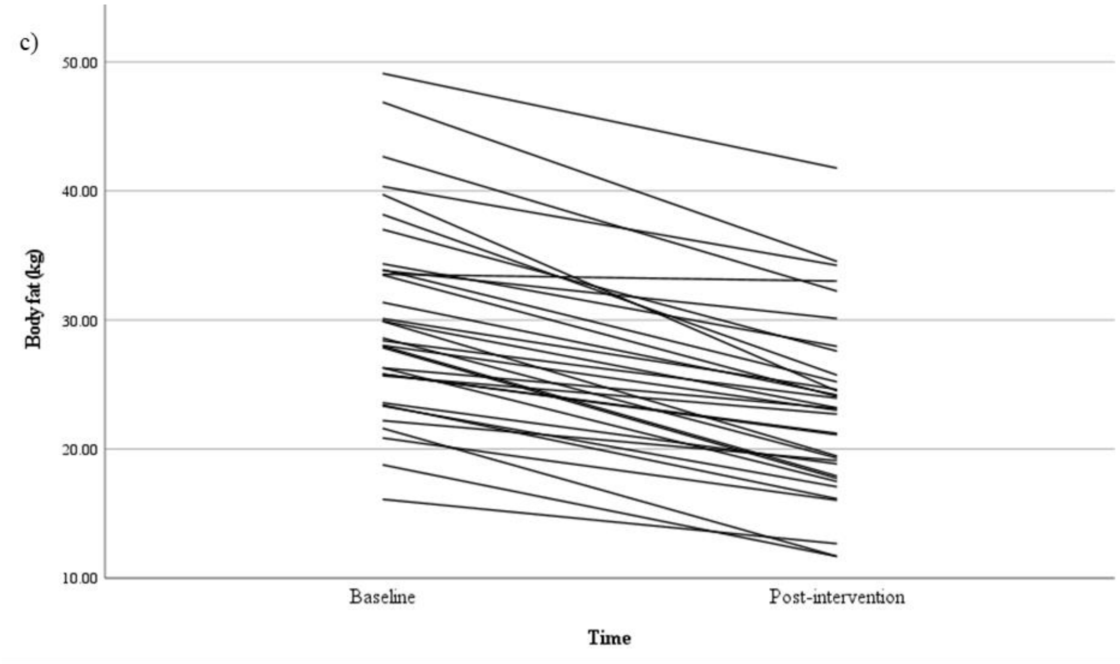

Key: Graphs show individual changes in a) weight; b) lean body mass and c) body fat.

Supplemental Table 1. Baseline participant characteristics between sexes

Baseline variables	IFT Males (n = 9)	CERT Males (n = 8)	*p*-value[1]	IFT Females (n = 8)	CERT Females (n = 9)	*p*-value[2]
Age (years)	25.2 (6.2)	23.1 (2.6)	0.39	24.1 (2.9)	23.2 (4.9)	0.66
Height (m)	1.81 (0.1)	1.79 (0.1)	0.51	1.62 (0.1)	1.64 (0.1)	0.42
Weight (kg)	87.3 (12.5)	88.0 (11.5)	0.91	71.9 (10.7)	72.1 (10.6)	0.98
BMI (kg/m^2)	26.6 (3.0)	27.6 (2.4)	0.48	27.5 (2.5)	26.7 (3.4)	0.60
LBM (kg)	64.4 (7.2)	64.6 (9.4)	0.96	43.0 (5.6)	43.2 (4.8)	0.92
Body Fat Percentage (%)	29.4 (6.0)	30.4 (3.7)	0.68	43.0 (3.9)	42.2 (6.3)	0.76
Bench press 3RM (kg)	58.0 (12.1)	57.0 (12.9)	0.87	26.8 (4.9)	23.9 (5.6)	0.28
Bench press volume (70% 1RM) (kg)	610.6 (197.0)	522.1 (127.6)	0.29	283.4 (104.1)	247.2 (66.3)	0.40
Leg Press 3RM (kg)	150.6 (37.1)	165.0 (47.9)	0.49	68.8 (32.8)	57.4 (22.8)	0.42
Leg press volume (70% 1RM) (kg)	1636.2 (781.6)	1468.3 (578.0)	0.63	759.4 (558.1)	582.0 (298.4)	0.42

Note: Mean (*SD*). [1]P-values reported are for independent t-tests between males in different intervention groups. [2]P-values reported are for independent t-tests between females in different intervention groups. BMI = body mass index, LBM = lean body mass, 1RM = 1 repetition maximum, 3RM = 3 repetition maximum.

Supplemental Table 2. Regional differences in LBM between intervention groups (DXA)

Region	Group	Baseline	Week 12	Δ	Δ (%)	*P* (group)	*P* (time)	*P* (I)
Upper Body (kg)	IFT (n=17)	31.7 (8.0)	32.9 (7.6)	1.2	3.8	0.90	<0.001*	0.42
	CERT (n=17)	31.6 (8.6)	32.0 (8.6)	0.4	1.3			
Lower Body (kg)	IFT (n=17)	18.6 (4.3)	19.5 (4.4)	0.9	4.8	0.65	<0.001*	0.99
	CERT (n=17)	17.9 (4.3)	18.8 (4.0)	0.9	5.0			

Note: Mean (*SD*). *Significantly different than baseline at week 12 in all groups combined. Group = main effect for diet group, I = time x group interaction, Time = main effect for time.
